# Supplementary material for: Multidimensional sleep profiles via machine learning and risk of dementia and cardiovascular disease
Source: Commun Med (Lond). 2025 Jul 22;5:306. doi: 10.1038/s43856-025-01019-x (PMC12283935; doi:10.1038/s43856-025-01019-x)
Supplement: Supplementary file 2 — Supplementary Information [file 43856_2025_1019_MOESM2_ESM.pdf]

Supplementary Information

Multidimensional Sleep Profiles via Machine learning and Risk of Dementia and Cardiovascular Disease

Clémence Cavaillès, PhD, Meredith Wallace, PhD, Yue Leng, PhD, Katie L. Stone, PhD, Sonia Ancoli-Israel, PhD, Kristine Yaffe, MD

Supplementary Table 1. Baseline sample characteristics between included and excluded men. 4

Supplementary Table 2. Variable loadings on the six principal components retained from the principal component analysis. 5

Supplementary Table 3. New profiles and sleep characteristics among the 2,630 participants without intermittent use of nightly mechanical devices during sleep. 6

Supplementary Table 4. Cox regression models of the association between sleep profiles and the incidence of dementia (n=2,562) and cardiovascular disease events (n=2,606). 7

Supplementary Table 5. Cox regression models of the association between sleep profiles and the incidence of dementia (n=2,424) and cardiovascular disease events (n=2,455) after adjusting for baseline apnea-hypopnea index (AHI). 8

Supplementary Table 6. Cox regression models of the association between sleep profiles and the incidence of dementia (n=2,548) and cardiovascular disease events (n=2,590) after adjusting for living alone. 8

Supplementary Table 7. Cox regression models of the association between sleep profiles and dementia incidence after adjusting for baseline Modified Mini-Mental State Examination (3MS) score (n=2,562). 9

Supplementary Table 8. Cox regression models of the association between sleep profiles and dementia incidence after exclusion of incident dementia cases identified at the first follow-up visit (n=2,403). 10

Supplementary Table 9. Cox regression models of the association between sleep profiles and the incidence of cardiovascular disease events after exclusion of participants with a history of heart attack or stroke (n=2,106). 10

Supplementary Figure 1. Flow chart. 11

Supplementary Figure 2. Heatmap. 12

Supplementary Figure 3. Criteria for determining the number of principal components. 13

Supplementary Figure 4. Distributions of actigraphy variables used to identify the sleep clusters.. 14

Supplementary Figure 5. Criteria for determining the number of clusters. 15

Supplementary Table 1. Baseline sample characteristics between included and excluded men.

	Included (n= 2,667)	Excluded (n=468)	
**Characteristics**	**Median (IQR) or No. (%)**	**Median (IQR) or No. (%)**	**p-value[a]**
Age (years)	75 (72;80)	77 (73;82)	5.57e-09
Education, ≤*High school*	539 (20.2)	127 (27.1)	0.0007
Race/ethnicity			4.64e-08
White	2425 (90.9)	391 (83.5)	
Black/African American	82 (3.1)	39 (8.3)	
Other	160 (6.0)	38 (8.1)	
Living alone	319 (12.0)	86 (18.4)	0.0001
PASE score	142.5 (97.2;187.2)	130.6 (88.5;184.1)	0.01
GDS score, ≥*6*	156 (5.9)	55 (11.8)	2.32e-06
Smoking status			0.46
Never	1054 (39.5)	181 (38.8)	
Past	1561 (58.6)	273 (58.5)	
Current	51 (1.9)	13 (2.8)	
Caffeine intake (mg/day)	184 (36;368)	144 (0;314)	0.045
Alcoholic drink per week, >*1*	1452 (54.7)	203 (43.8)	1.27e-05
BMI	26.8 (24.6;29.4)	26.2 (24.3;29.4)	0.18
History of heart attack	450 (16.9)	90 (19.3)	0.21
History of stroke	93 (3.5)	24 (5.1)	0.08
History of diabetes mellitus	352 (13.2)	65 (13.9)	0.67
History of hypertension	1324 (49.7)	236 (50.5)	0.73
Current sleep medication, ≥*1*	304 (11.4)	89 (19.1)	4.02e-06
Antidepressants	187 (7.0)	61 (13.1)	7.89e-06
BZD	107 (4.0)	32 (6.9)	0.006
Other sleep medications	50 (1.9)	12 (2.6)	0.32

Abbreviations: BZD, benzodiazepine; BMI, body mass index; GDS, Geriatric Depression Scale; IQR, interquartile range; PASE, Physical Activity Scale for the Elderly.

[a] Kruskal-Wallis test was used for continuous variables, Chi-square test for categorical variables

Supplementary Table 2. Variable loadings on the six principal components retained from the principal component analysis.

	**PC1**	**PC2**	**PC3**	**PC4**	**PC5**	**PC6**
Acrophase	0.293	-0.304	0.150	-0.157	0.142	-0.093
Alpha	0.031	0.040	-0.133	0.483	-0.481	0.195
Beta	0.064	0.186	0.062	0.072	0.317	0.352
Interdaily stability (IS)	-0.257	-0.292	-0.207	-0.015	0.130	0.252
Intradaily variability (IV)	0.196	0.355	0.220	0.042	0.138	0.120
Mesor	-0.052	-0.132	-0.470	0.078	-0.344	0.147
Midpoint of L5	0.357	-0.224	0.017	-0.008	-0.034	0.233
Minimum	0.167	0.244	-0.286	0.182	0.073	0.216
Minutes Napping	0.129	0.326	0.229	0.132	-0.316	0.167
Pseudo-F	-0.219	-0.343	-0.228	-0.030	0.006	0.028
SD Sleep Onset	0.217	0.101	-0.236	0.058	-0.045	-0.384
SD Wake-Up Time	0.185	0.018	0.031	-0.013	-0.349	-0.493
Sleep Latency	0.199	0.081	-0.329	0.059	0.245	-0.168
Sleep Onset Time	0.369	-0.082	-0.098	-0.266	-0.111	0.151
Start of L5	0.357	-0.224	0.017	-0.008	-0.034	0.233
Start of M10	0.246	-0.261	0.101	0.014	-0.061	-0.157
Time in Bed	-0.009	-0.151	0.064	0.598	0.279	-0.212
Total Sleep Duration	-0.175	-0.231	0.384	0.351	-0.053	-0.027
Wake After Sleep Onset	0.155	0.115	-0.329	0.223	0.315	-0.138
Wake-Up Time	0.287	-0.278	0.112	0.262	0.075	0.120

Supplementary Table 3. New profiles and sleep characteristics among the 2,630 participants without intermittent use of nightly mechanical devices during sleep.

	**Active Healthy Sleepers** (n=1,450)	**Fragmented Poor Sleepers** (n=591)	**Long and Frequent Nappers** (n=588)
**Sleep variables**	**Median (IQR)**	**Median (IQR)**	**Median (IQR)**
**Variables used to create the clusters**			
Alpha[a]	-0.41 (-0.50;-0.30)	-0.35 (-0.46;-0.21)	-0.09 (-0.25;0.10)
Minimum[a]	210 (0;349)	499 (308;725)	540 (293;728)
Wake After Sleep Onset	58 (41;78)	119 (91;150)	60 (39;86)
Minutes Napping	34 (16;60)	31 (15;56)	82 (48;132)
Sleep Latency	17 (11;27)	47 (28;77)	18 (11;29)
SD Sleep Onset	0.55 (0.35;0.78)	1.07 (0.72;1.52)	0.52 (0.33;0.78)
Acrophase[a]	14.33 (13.72;14.91)	14.81 (13.97;15.68)	13.50 (12.87;14.14)
Total Sleep Duration	403 (367;441)	344 (280;398)	391 (358;429)
Pseudo-F[a]	1082 (792;1400)	921 (653;1310)	823 (597;1135)
Time in Bed	487 (451;523)	517 (477;557)	478 (443;514)
Start of M10[b]	8.1 (7.2;9.1)	8.8 (7.8;10.0)	7.6 (6.8;8.5)
Sleep Onset Time	23.0 (22.4;23.6)	23.8 (22.9;24.8)	23.3 (22.7;24.0)
Intradaily variability (IV)[b]	0.59 (0.48;0.71)	0.62 (0.49;0.77)	0.69 (0.55;0.85)
Wake-Up Time	6.8 (6.2;7.4)	7.6 (6.8;8.2)	6.9 (6.3;7.5)
SD Wake-Up Time	0.56 (0.37;0.79)	0.70 (0.45;1.02)	0.46 (0.29;0.73)
Interdaily stability (IS)[b]	0.76 (0.69;0.82)	0.73 (0.65;0.80)	0.74 (0.67;0.80)
Mesor[a]	2073 (1837;2318)	2237 (1966;2539)	2243 (1879;2617)
Start of L5[b]	0.07 (-0.82;0.88)	1.27 (0.11;2.08)	0.42 (-0.37;1.27)
Midpoint of L5[b]	2.57 (1.68;3.38)	3.77 (2.61;4.58)	2.92 (2.13;3.77)
Beta[a]	7.96 (4.82;16.86)	8.63 (5.18;18.71)	12.40 (4.32;40.20)
**Other variables**			
Sleep Efficiency	84 (79;88)	67 (58;75)	83 (76;88)
Down-Mesor[a]	21.9 (21.1;22.7)	22.1 (21.1;23.3)	19.9 (18.9;20.9)
Sleep Maintenance	87 (83;91)	73 (66;80)	87 (81;91)
Relative amplitude (RA)[b]	0.87 (0.83;0.90)	0.78 (0.71;0.83)	0.85 (0.79;0.89)
Number of Naps	2.5 (1.3;4.0)	2.3 (1.0;4.0)	5.5 (3.5;9.0)
L5[b]	278 (210;361)	493 (397;641)	299 (219;415)
SD Midpoint (Onset interval)	0.43 (0.30;0.62)	0.72 (0.50;0.98)	0.40 (0.27;0.60)
Midpoint of M10[b]	13.1 (12.2;14.1)	13.8 (12.8;15.0)	12.6 (11.8;13.5)
Midpoint (Onset Interval)	2.83 (2.30;3.37)	3.55 (2.92;4.38)	3.04 (2.55;3.63)
Amplitude[a]	3702 (3159;4260)	3436 (2732;4142)	3284 (2640;4155)
Up-Mesor[a]	6.8 (6.2;7.4)	7.6 (6.8;8.3)	7.0 (6.4;7.8)
SD Midpoint (Bed interval)	0.43 (0.29;0.60)	0.57 (0.38;0.82)	0.37 (0.24;0.57)
M10[b]	4039 (3540;4521)	4006 (3489;4606)	3703 (3132;4328)
SD Bed Time	0.50 (0.32;0.74)	0.69 (0.44;1.03)	0.46 (0.28;0.72)
Bed Time	22.6 (22.0;23.3)	22.8 (22.0;23.7)	22.9 (22.3;23.6)
Midpoint (Bed Interval)	2.71 (2.18;3.23)	3.11 (2.47;3.90)	2.91 (2.43;3.48)
Time from Onset to Wake-Up	461 (423;495)	451 (397;502)	449 (414;486)

Supplementary Table 4. Cox regression models of the association between sleep profiles and the incidence of dementia (n=2,562) and cardiovascular disease events (n=2,606).

	Unadjusted model		Model 1		Model 2	
	HR (95% CI)	p	HR (95% CI)	p	HR (95% CI)	p
Dementia		0.09		0.11		0.08
Active healthy sleepers	1		1		1	
Fragmented poor sleepers	1.34 (1.03;1.74)	0.03	1.35 (1.02;1.78)	0.03	1.39 (1.04;1.85)	0.02
Long and frequent nappers	1.11 (0.89;1.39)	0.37	1.09 (0.86;1.38)	0.48	1.11 (0.87;1.42)	0.41
Cardiovascular disease events		0.0004		0.01		0.007
Active healthy sleepers	1		1		1	
Fragmented poor sleepers	1.44 (1.19;1.74)	0.0002	1.32 (1.08;1.60)	0.006	1.34 (1.10;1.63)	0.003
Long and frequent nappers	1.21 (1.02;1.42)	0.02	1.16 (0.98;1.37)	0.08	1.18 (1.00;1.39)	0.05

Age was used as time scale. Model 1 was adjusted for site, race/ethnicity, education, smoking status, caffeine intake, alcohol use, physical activity, body mass index, history of diabetes mellitus and hypertension, depressive symptoms, and sleep-related medications use. Model 2 was further adjusted for history of heart attack and stroke.

Supplementary Table 5. Cox regression models of the association between sleep profiles and the incidence of dementia (n=2,424) and cardiovascular disease events (n=2,455) after adjusting for baseline apnea-hypopnea index (AHI).

	Model 1		Model 2	
	HR (95% CI)	p	HR (95% CI)	p
Dementia		0.15		0.11
Active healthy sleepers	1		1	
Fragmented poor sleepers	1.33 (1.00;1.78)	0.05	1.38 (1.02;1.86)	0.04
Long and frequent nappers	1.11 (0.87;1.41)	0.41	1.14 (0.88;1.47)	0.31
Cardiovascular disease events		0.006		0.003
Active healthy sleepers	1		1	
Fragmented poor sleepers	1.38 (1.13;1.69)	0.001	1.41 (1.15;1.72)	0.0008
Long and frequent nappers	1.15 (0.97;1.37)	0.11	1.17 (0.99;1.40)	0.07

Age was used as time scale. Model 1 was adjusted for site, race/ethnicity, education, smoking status, caffeine intake, alcohol use, physical activity, body mass index, history of diabetes mellitus and hypertension, depressive symptoms, sleep-related medications use, and baseline AHI. Model 2 was further adjusted for history of heart attack and stroke.

Supplementary Table 6. Cox regression models of the association between sleep profiles and the incidence of dementia (n=2,548) and cardiovascular disease events (n=2,590) after adjusting for living alone.

	Model 1		Model 2	
	HR (95% CI)	p	HR (95% CI)	p
Dementia		0.12		0.08
Active healthy sleepers	1		1	
Fragmented poor sleepers	1.35 (1.02;1.78)	0.03	1.39 (1.05;1.85)	0.02
Long and frequent nappers	1.09 (0.86;1.38)	0.49	1.11 (0.87;1.42)	0.41
Cardiovascular disease events		0.02		0.009
Active healthy sleepers	1		1	
Fragmented poor sleepers	1.30 (1.07;1.59)	0.007	1.33 (1.09;1.62)	0.004
Long and frequent nappers	1.16 (0.98;1.37)	0.08	1.18 (1.00;1.40)	0.05

Age was used as time scale. Model 1 was adjusted for site, race/ethnicity, education, smoking status, caffeine intake, alcohol use, physical activity, body mass index, history of diabetes mellitus and hypertension, depressive symptoms, sleep-related medications use, and living alone. Model 2 was further adjusted for history of heart attack and stroke.

Supplementary Table 7. Cox regression models of the association between sleep profiles and dementia incidence after adjusting for baseline Modified Mini-Mental State Examination (3MS) score (n=2,562).

	Model 1		Model 2	
	HR (95% CI)	p	HR (95% CI)	p
Dementia		0.16		0.11
Active healthy sleepers	1		1	
Fragmented poor sleepers	1.32 (1.00;1.74)	0.049	1.37 (1.03;1.82)	0.03
Long and frequent nappers	1.07 (0.84;1.35)	0.58	1.09 (0.85;1.40)	0.49

Age was used as time scale. Model 1 was adjusted for site, race/ethnicity, education, smoking status, caffeine intake, alcohol use, physical activity, body mass index, history of diabetes mellitus and hypertension, depressive symptoms, sleep-related medications use, and baseline 3MS score. Model 2 was further adjusted for history of heart attack and stroke.

Supplementary Table 8. Cox regression models of the association between sleep profiles and dementia incidence after exclusion of incident dementia cases identified at the first follow-up visit (n=2,403).

	Unadjusted model		Model 1		Model 2	
	HR (95% CI)	p	HR (95% CI)	p	HR (95% CI)	p
Dementia		0.10		0.10		0.11
Active healthy sleepers	1		1		1	
Fragmented poor sleepers	1.41 (1.03;1.95)	0.03	1.42 (1.01;1.98)	0.04	1.41 (1.00,1.97)	0.048
Long and frequent nappers	1.17 (0.89;1.54)	0.26	1.20 (0.90;1.61)	0.20	1.20 (0.90,1.61)	0.21

Age was used as time scale. Model 1 was adjusted for site, race/ethnicity, education, smoking status, caffeine intake, alcohol use, physical activity, body mass index, history of diabetes mellitus and hypertension, depressive symptoms, and sleep-related medications use. Model 2 was further adjusted for history of heart attack and stroke.

Supplementary Table 9. Cox regression models of the association between sleep profiles and the incidence of cardiovascular disease events after exclusion of participants with a history of heart attack or stroke (n=2,106).

	Unadjusted model		Model 1	
	HR (95% CI)	p	HR (95% CI)	p
Cardiovascular disease events		0.003		0.04
Active healthy sleepers	1		1	
Fragmented poor sleepers	1.48 (1.19;1.85)	0.0004	1.36 (1.08;1.70)	0.009
Long and frequent nappers	1.12 (0.91;1.36)	0.28	1.07 (0.87;1.31)	0.51

Age was used as time scale. Model 1 was adjusted for site, race/ethnicity, education, smoking status, caffeine intake, alcohol use, physical activity, body mass index, history of diabetes mellitus and hypertension, depressive symptoms, and sleep-related medications use. Model 2 was further adjusted for history of heart attack and stroke.

Supplementary Figure 1. Flow chart.

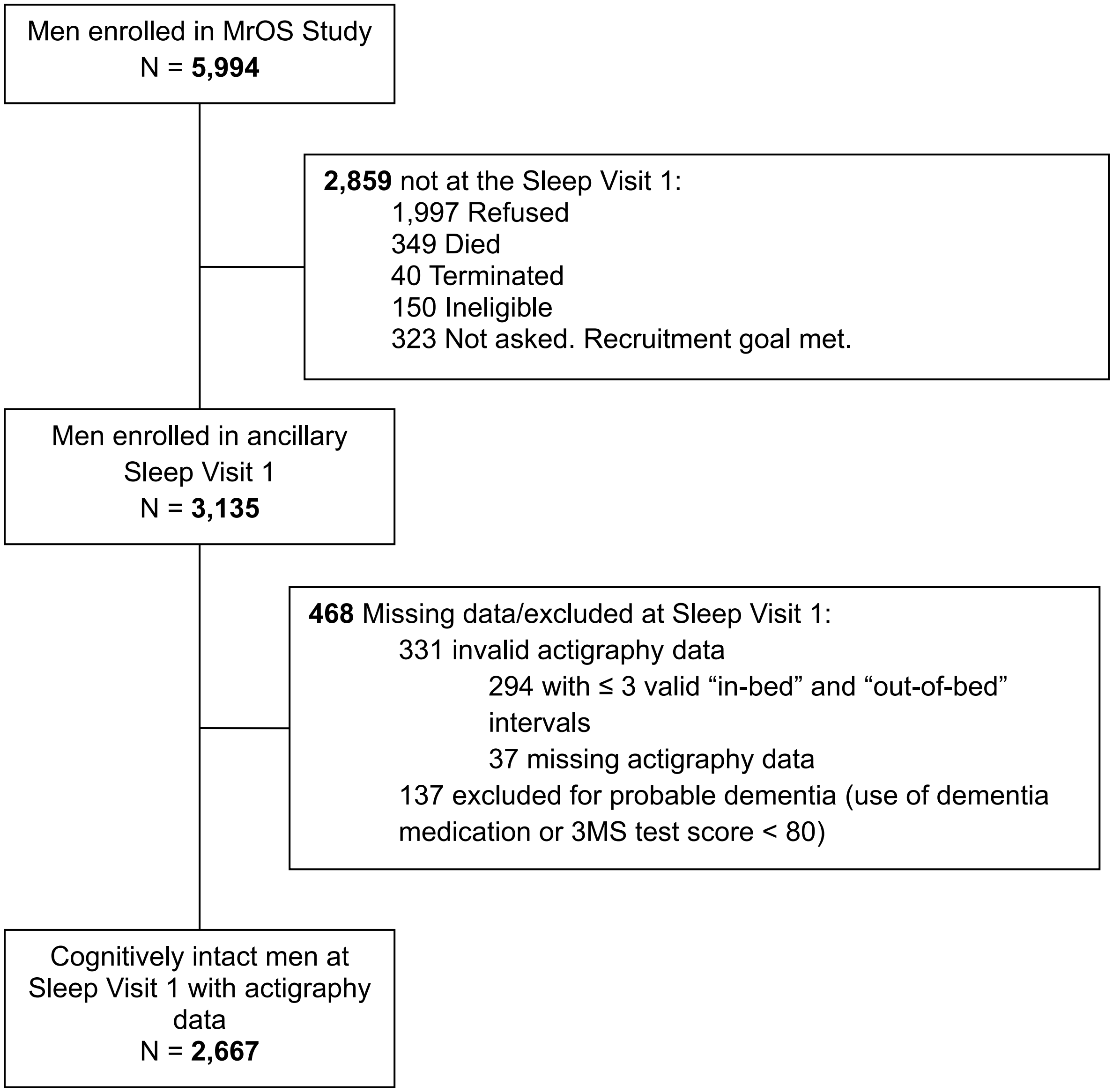

Supplementary Figure 2. Heatmap.

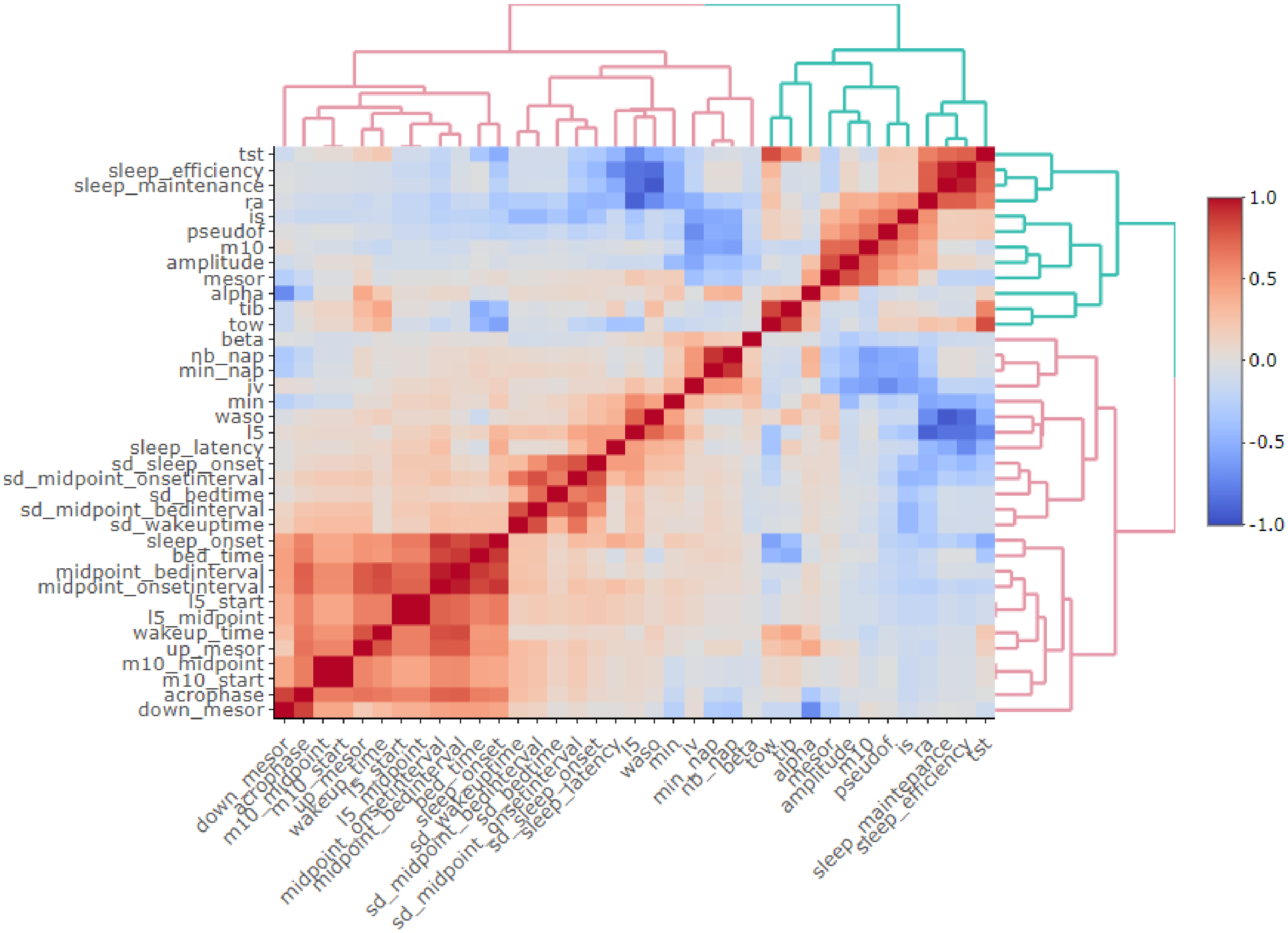

The heatmap displays the Pearson correlation matrix for all actigraphy-derived variables. The color gradient indicates the strength and direction of correlations, ranges from blue (negative correlations) to red (positive correlations).

Supplementary Figure 3. Criteria for determining the number of principal components.

A) Principal component description

Loadings	Eigenvalue	Variance explained (%)	Cumulative variance explained (%)
**PC1**	4.72	23.6	23.6
**PC2**	3.23	16.1	39.7
**PC3**	2.40	12.0	51.7
**PC4**	2.01	10.1	61.8
**PC5**	1.42	7.1	68.9
**PC6**	1.35	6.7	75.6
**PC7**	0.89	4.5	80.1
**PC8**	0.75	3.8	83.9
**PC9**	0.66	3.3	87.2
**PC10**	0.47	2.4	89.5
**PC11**	0.44	2.2	91.7
**PC12**	0.39	2.0	93.7
**PC13**	0.38	1.9	95.6
**PC14**	0.33	1.6	97.2
**PC15**	2.61	1.3	98.5
**PC16**	1.70	0.8	99.4
**PC17**	0.10	0.5	99.9
**PC18**	0.02	0.1	100.0
**PC19**	0.01	0.0	100.0
**PC20**	0.00	0.0	100.0

B) Scree plot

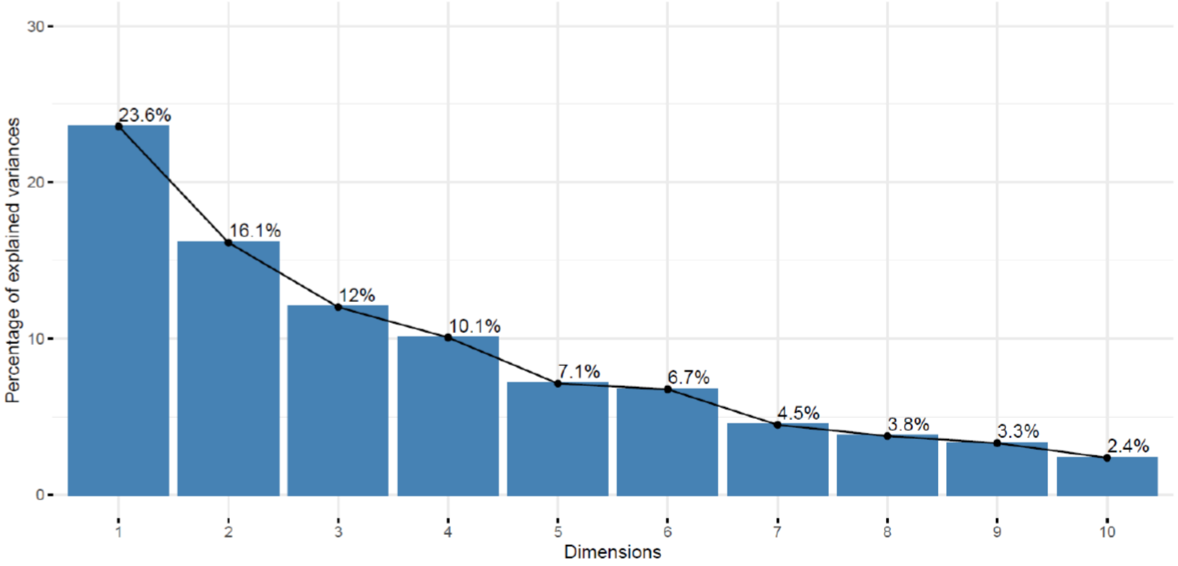

Abbreviations: PC, principal component.

Panel A represent the loadings, eigenvalue, variance explained (%), and cumulative variance explained (%) of the principal components. Panel B display the percentage of explained variances for principal component 1 to 10.

Supplementary Figure 4. Distributions of actigraphy variables used to identify the sleep clusters.

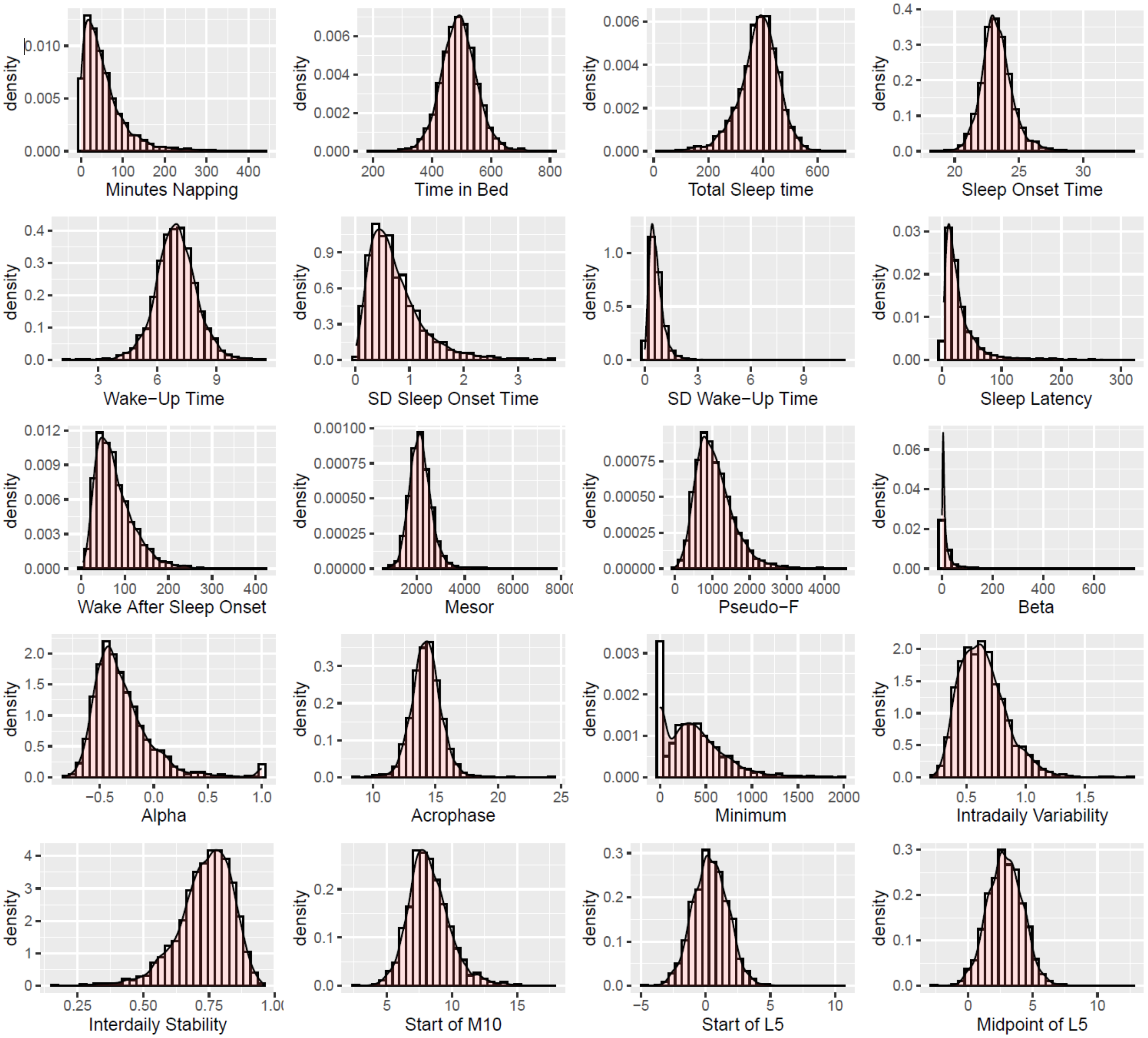

These 20 panels represent the distribution of each variable used to identify the sleep profiles.

Supplementary Figure 5. Criteria for determining the number of clusters.

A) AIC criteria

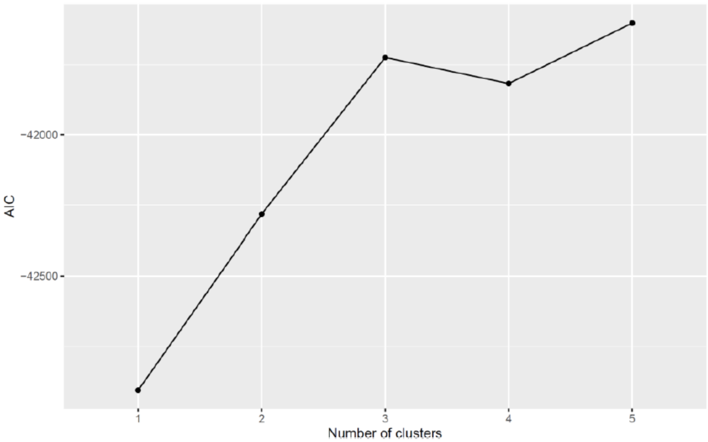

B) BIC criteria

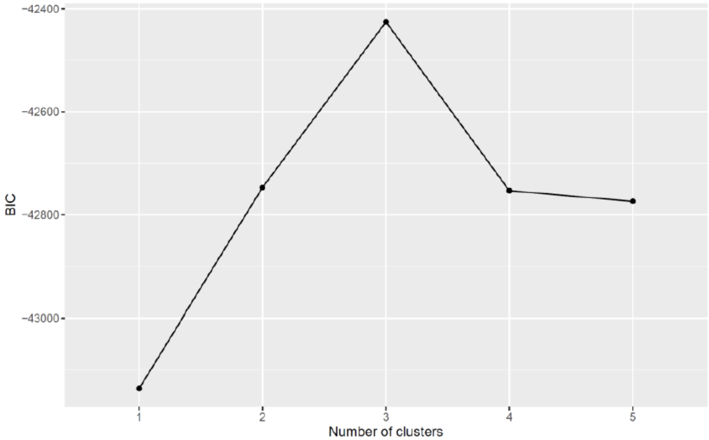

C) ICL criteria

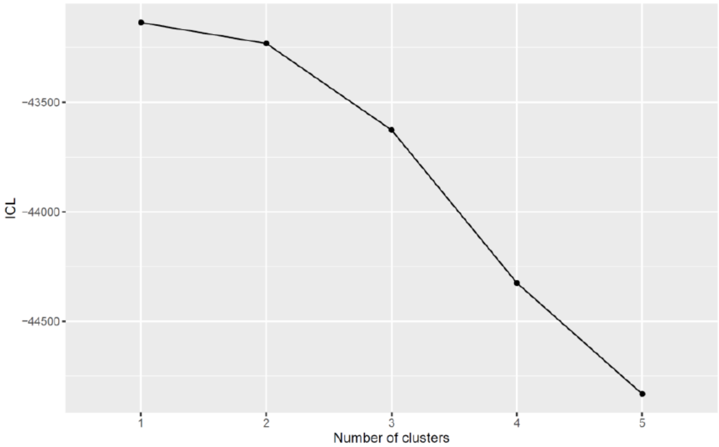

D) Summary of all criteria

Number of clusters	BIC	AIC	ICL	Number of elements
1	-43136.21	-42906.55	-43136.21	2667
2	-42747.15	-42281.94	-43231.11	99; 2568
3	-42425.30	-41724.54	-41843.54	1707; 376; 584
4	-42743.44	-41817.14	-44324.59	319; 1503; 324; 521
5	-42773.93	-41602.07	-41801.07	619; 600; 581; 545; 322

Abbreviations: AIC, Akaike Information Criteria; BIC, Bayesian Information Criteria; ICL, Integrated Complete-data Likelihood.

Panels A, B, and C show the AIC, the BIC, and the ICL values, respectively, when considering 1 to 5 clusters. Panel D provides a summary of all three criteria across the 1- to 5-cluster solutions, along with the number of participants in each cluster.
