# Supplementary material for: Multidimensional sleep profiles via machine learning and risk of dementia and cardiovascular disease
Source: Commun Med (Lond). 2025 Jul 22;5:306. doi: 10.1038/s43856-025-01019-x (PMC12283935; doi:10.1038/s43856-025-01019-x)
Supplement: Supplementary file 3 — Description of Additional Supplementary Files [file 43856_2025_1019_MOESM3_ESM.pdf]

Description of Additional Supplementary Files

File name: Supplementary Data 1-2

Description: Source data underlying figures 1-2
