# Supplementary material for: Multidimensional sleep profiles via machine learning and risk of dementia and cardiovascular disease
Source: Commun Med (Lond). 2025 Jul 22;5:306. doi: 10.1038/s43856-025-01019-x (PMC12283935; doi:10.1038/s43856-025-01019-x)
Supplement: Supplementary file 6 — Reporting Summary [file 43856_2025_1019_MOESM6_ESM.pdf]

nature portfolio

Corresponding author(s): CLEMENCE CAVAILLES

Last updated by author(s): Jul 2, 2025

Reporting Summary

Nature Portfolio wishes to improve the reproducibility of the work that we publish. This form provides structure for consistency and transparency in reporting. For further information on Nature Portfolio policies, see our Editorial Policies and the Editorial Policy Checklist.

Statistics

For all statistical analyses, confirm that the following items are present in the figure legend, table legend, main text, or Methods section.

n/a	Confirmed	
☐	☒	The exact sample size (*n*) for each experimental group/condition, given as a discrete number and unit of measurement
☐	☒	A statement on whether measurements were taken from distinct samples or whether the same sample was measured repeatedly
☐	☒	The statistical test(s) used AND whether they are one- or two-sided<br>*Only common tests should be described solely by name; describe more complex techniques in the Methods section.*
☐	☒	A description of all covariates tested
☐	☒	A description of any assumptions or corrections, such as tests of normality and adjustment for multiple comparisons
☐	☒	A full description of the statistical parameters including central tendency (e.g. means) or other basic estimates (e.g. regression coefficient) AND variation (e.g. standard deviation) or associated estimates of uncertainty (e.g. confidence intervals)
☐	☒	For null hypothesis testing, the test statistic (e.g. *F*, *t*, *r*) with confidence intervals, effect sizes, degrees of freedom and *P* value noted<br>*Give P values as exact values whenever suitable.*
☒	☐	For Bayesian analysis, information on the choice of priors and Markov chain Monte Carlo settings
☐	☒	For hierarchical and complex designs, identification of the appropriate level for tests and full reporting of outcomes
☐	☒	Estimates of effect sizes (e.g. Cohen's *d*, Pearson's *r*), indicating how they were calculated

Our web collection on statistics for biologists contains articles on many of the points above.

Software and code

Policy information about availability of computer code

Data collection	NA
Data analysis	Statistical analyses were performed using R version 4.3.0.

For manuscripts utilizing custom algorithms or software that are central to the research but not yet described in published literature, software must be made available to editors and reviewers. We strongly encourage code deposition in a community repository (e.g. GitHub). See the Nature Portfolio guidelines for submitting code & software for further information.

Data

Policy information about availability of data

All manuscripts must include a data availability statement. This statement should provide the following information, where applicable:

- Accession codes, unique identifiers, or web links for publicly available datasets
- A description of any restrictions on data availability
- For clinical datasets or third party data, please ensure that the statement adheres to our policy

The data supporting the findings of this study are openly available at https://mrosonline.ucsf.edu.

Research involving human participants, their data, or biological material

Policy information about studies with human participants or human data. See also policy information about sex, gender (identity/presentation), and sexual orientation and race, ethnicity and racism.

Reporting on sex and gender	The Osteoporotic Fractures in Men Study (MrOS) enrolled 5,994 community-dwelling men aged ≥65 years. Therefore, our study includes only men participants. This is specified throughout the entire manuscript.
Reporting on race, ethnicity, or other socially relevant groupings	Race and ethnicity were self-reported at baseline. This variable was used as a potential confounding factor and controlled for in the multivariate analyses. This variable include White, Black/African American and Other participants as described in the tables.
Population characteristics	Population characteristics are described in Supplementary Table 1, in Table 3 by sleep profiles, and in the Results section. A total of 2,667 men were eligible for cluster analysis. At baseline, participants had a median age of 75 years (interquartile range [IQR]= 72-80), 20.2% had a high school education or lower, and 90.0% were White. Compared to included participants, excluded men (n=468) were older, less educated, more likely to be non-White and to live alone, and had less physical activity and alcohol consumption, but higher depressive symptoms and sleep medication use (Supplementary Table 1). Compared to AHS, FPS were less educated and less physically active, while LFN were slightly older. Both FPS and LFN were more likely to be non-White, smokers, to have a history of hypertension and a higher BMI. AHS consumed less caffeine than FPS (Table 3).
Recruitment	From 2000 to 2002, the Osteoporotic Fractures in Men Study (MrOS) enrolled 5,994 community-dwelling men aged ≥65 years, able to walk without assistance, and without bilateral hip replacements, at six clinical centers across the United States15,16. Among them, 3,135 were recruited into the ancillary MrOS sleep study and underwent a comprehensive sleep assessment between 2003 and 2005 (our study baseline). Men were screened for use of mechanical devices, including pressure masks for sleep apnea (positive airway pressure or oral appliance devices), or nocturnal oxygen therapy, and were excluded if they reported nightly use of any of these devices (except for intermittent users, n=49). We excluded 331 men with missing actigraphy data or with fewer than 3 days or fewer than 3 nights of actigraphy data, and 137 with significant cognitive impairment at baseline (Modified Mini-Mental State Examination (3MS) score <80 or taking dementia medication), leading to a sample of 2,667 participants (Supplementary Fig. 1). This study predominantly involves White older men, limiting the generalizability of the results. Future research should replicate these methods in more diverse samples.
Ethics oversight	All participants provided written informed consent and the study was approved by the Institutional Review Board at each site. Our analytic study was approved by University of California San Francisco IRB.

Note that full information on the approval of the study protocol must also be provided in the manuscript.

Field-specific reporting

Please select the one below that is the best fit for your research. If you are not sure, read the appropriate sections before making your selection.

☐ Life sciences ☒ Behavioural & social sciences ☐ Ecological, evolutionary & environmental sciences

For a reference copy of the document with all sections, see nature.com/documents/nr-reporting-summary-flat.pdf

Behavioural & social sciences study design

All studies must disclose on these points even when the disclosure is negative.

Study description	Prospective observational study
Research sample	From 2000 to 2002, the Osteoporotic Fractures in Men Study (MrOS) enrolled 5,994 community-dwelling men aged ≥65 years, able to walk without assistance, and without bilateral hip replacements, at six clinical centers across the United States15,16. Among them, 3,135 were recruited into the ancillary MrOS sleep study and underwent a comprehensive sleep assessment between 2003 and 2005 (our study baseline). Men were screened for use of mechanical devices, including pressure masks for sleep apnea (positive airway pressure or oral appliance devices), or nocturnal oxygen therapy, and were excluded if they reported nightly use of any of these devices (except for intermittent users, n=49). We excluded 331 men with missing actigraphy data or with fewer than 3 days or fewer than 3 nights of actigraphy data, and 137 with significant cognitive impairment at baseline (Modified Mini-Mental State Examination (3MS) score <80 or taking dementia medication), leading to a sample of 2,667 participants (Supplementary Fig. 1). This study predominantly involves White older men, limiting the generalizability of the results. Future research should replicate these methods in more diverse samples.
Sampling strategy	No sample-size calculation was performed. This study included 2,667 participants which is large compared to most prior studies in this context.
Data collection	Participants wore a SleepWatch-O actigraph (Ambulatory Monitoring, Inc) continuously on their nondominant wrist for ≥4 consecutive 24-hours periods. Data were collected in proportional integration mode and scored by epoch to estimate “wake” and “sleep” periods using Action W-2 software and the University of California, San Diego scoring algorithm17. Trained scorers at the San Francisco Coordinating Center edited the data using participants’ sleep diaries to identify time in and out of bed as well as periods when the interval should be deleted because the watch was removed. Over 12 years, participants attended four follow-up visits where they reported any physician-diagnosed dementia and their medication use, bringing all medications taken within the past 30

days. Dementia medication use was categorized based on the Iowa Drug Information Service Drug Vocabulary22. In addition, trained staff administered the 3MS test to assess global cognitive function. Incident dementia at any follow-up visit was defined by meeting at least one of the following criteria: (i) self-reported physician-diagnosed dementia; (ii) dementia medication use; or (iii) a change in 3MS score of ≥1.5 SDs worse than the mean change from baseline to any follow-up visit. Participants were censored at the date of the diagnostic visit, death, or last visit. Participants were surveyed for incident CVD events by postcard and phone contact every four months for approximately 12 years, with a response rate over 99%. Relevant medical records and documentation from any potential incident clinical events were obtained by the clinical center. For both nonfatal and fatal CVD events, all documents were adjudicated by a board-certified cardiologist using a prespecified adjudication protocol. Inter-rater agreement was periodically evaluated by one or more expert adjudicator(s) in a random subset of events to ensure quality control.

Timing

Actigraphy data was obtained at baseline. Participants were censored at the date of the diagnostic visit, death, or last visit for dementia incidence. Participants were censored at the date of the first cardiovascular disease event, death, last contact before March 1, 2015, or on March 1, 2015 for cardiovascular disease event incidence.

Data exclusions

No data were excluded.

Non-participation

We excluded 331 men with missing actigraphy data or with fewer than 3 days or fewer than 3 nights of actigraphy data, and 137 with significant cognitive impairment at baseline (Modified Mini-Mental State Examination (3MS) score <80 or taking dementia medication), leading to a sample of 2,667 participants (Supplementary Fig. 1).

Randomization

Participants were not allocated to specific groups, no randomization was done.

Reporting for specific materials, systems and methods

We require information from authors about some types of materials, experimental systems and methods used in many studies. Here, indicate whether each material, system or method listed is relevant to your study. If you are not sure if a list item applies to your research, read the appropriate section before selecting a response.

Materials & experimental systems

n/a	Involved in the study
☒	☐ Antibodies
☒	☐ Eukaryotic cell lines
☒	☐ Palaeontology and archaeology
☒	☐ Animals and other organisms
☒	☐ Clinical data
☒	☐ Dual use research of concern
☒	☐ Plants

Methods

n/a	Involved in the study
☒	☐ ChIP-seq
☒	☐ Flow cytometry
☒	☐ MRI-based neuroimaging

Plants

Seed stocks

NA

Novel plant genotypes

NA

Authentication

NA
